# Video-based communication assessment for weight management counseling training in medical residents: a mixed methods study

**DOI:** 10.1186/s12909-022-03984-6

**Published:** 2022-12-28

**Authors:** Jamie M. Faro, Angelo D’Addario, Ann M. King, Kathleen M. Mazor, Lori Pbert, Rajani S. Sadasivam, Alan C. Geller, Elizabeth A. Murphy, Judith K. Ockene

**Affiliations:** 1 Department of Population and Quantitative Health Sciences, University of Massachusetts Chan Medical School, 368 Plantation St, Worcester, MA 01605 USA; 2 National Board of Medical Examiners, Philadelphia, USA; 3 Meyers Health Care Institute, University of Massachusetts Medical School, 385 Grove St, Worcester, MA USA; 4 Department of Medicine, University of Massachusetts Chan Medical School, 55 Lake Ave. North, Worcester, MA USA; 5 Department of Social and Behavioral Sciences, Harvard University, 677 Huntington Ave, Cambridge, MA USA

**Keywords:** Feedback, Analog patients, Residents, Weight management, Technology

## Abstract

**Background:**

Physician delivered weight management counseling (WMC) occurs infrequently and physicians report lack of training and poor self-efficacy. The purpose of this study was to develop and test the Video-based Communication Assessment (VCA) for weight management counseling (WMC) training in medical residents.

**Methods:**

This study was a mixed methods pilot conducted in 3 phases. First, we created five vignettes based on our prior data and expert feedback, then administered the vignettes via the VCA to Internal Medicine categorical residents (*n* = 16) from a University Medical School. Analog patients rated responses and also provided comments. We created individualized feedback reports which residents were able to view on the VCA. Lastly, we conducted debriefing interviews with the residents (*n* = 11) to obtain their feedback on the vignettes and personalized feedback. Interviews were transcribed, and we used thematic analysis to generate and apply codes, followed by identifying themes.

**Results:**

Descriptive statistics were calculated and learning points were created for the individualized feedback reports. In VCA debriefing interviews with residents, five themes emerged: 1) Overall the VCA was easy to use, helpful and more engaging than traditional learning and assessment modes, 2) Patient scenarios were similar to those encountered in the clinic, including diversity, health literacy and different stages of change, 3) The knowledge, skills, and reminders from the VCA can be transferred to practice, 4) Feedback reports were helpful, to the point and informative, including the exemplar response of how to best respond to the scenario, and 5) The VCA provide alternatives and practice scenarios to real-life patient situations when they aren’t always accessible.

**Conclusions:**

We demonstrated the feasibility and acceptability of the VCA, a technology delivered platform, for delivering WMC to residents. The VCA exposed residents to diverse patient experiences and provided potential opportunities to tailor providers responses to sociological and cultural factors in WMC scenarios. Future work will examine the effect of the VCA on WMC in actual clinical practice.

**Supplementary Information:**

The online version contains supplementary material available at 10.1186/s12909-022-03984-6.

## Background

Currently, two-thirds of US adults are living with overweight or obesity. It is estimated that by 2030, half of Americans will have obesity, reflecting an adult population that is transitioning from overweight to obesity [1]. Weight management counseling (WMC) by physicians during routine care has shown to result in increased patient motivation, physical activity, improved diet, and clinically significant weight loss [2, 3]. Weight loss has been significantly associated with improved health outcomes and a reduction in mortality risk [4, 5]. Unfortunately, rates of WMC in primary care are suboptimal [6], and physicians report a lack of WMC training and poor self-efficacy as barriers to providing WMC to their patients [7–9]. WMC is challenging, as physicians must discuss complex topics (e.g., weight history, diet, physical activity), support diverse patients, and refer patients to appropriate programs. Thus training across the medical education continuum is recommended [10–13]. Curriculum guidelines, competencies and learning objectives for WMC have been recommended by the Association of American Medical Colleges [14], but integration into curriculum has been limited [15]. Residency is a key time in physicians training as they assume major patient care responsibilities and learn critical skills in ambulatory practice [16, 17]. However, residents are unprepared to provide WMC and require a comprehensive evidence-based program [17–19].

Integrating a new training program within the residency curriculum is challenging. Residents’ schedules are complex as they divide their time between providing care at the hospital and ambulatory setting. The learning needs of medical trainees also change over the course of training. While the focus is on foundational training in medical school, it shifts towards experiential learning during residency. The Video-based Communication Assessment (VCA) is an innovative system providing experiential learning through practice and feedback developed by the National Board of Medical Examiners (NBME) [20–23]. The VCA presents the learner with brief vignettes depicting various patient scenarios and captures learners’ spoken communication (i.e., their actual words and voice tone) in response to vignettes. Accessible via smartphone application or web browser, the VCA allows learners to practice and receive feedback on their communication with patients in their own time. Thus, the VCA may be well-suited to support the learning needs of the residents.

In this paper we describe the development and testing of patient vignettes in the VCA regarding WMC for medical residents and subsequently providing formative feedback on their skill. We also examined the acceptability and practical application of the VCA system from the point of view of medical residents following use of the system.

## Methods

### Design

**Fig. 1:**
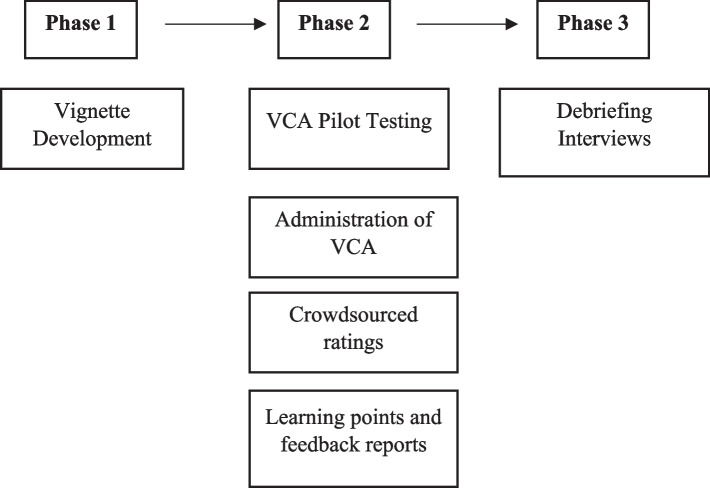
Overall study design by phase

This study was a mixed methods pilot conducted in 3 phases (see Fig. 1). The study team consisted of medical school researchers (JF, KM, LP, RS, JO), NBME researchers (AD, AK) and one clinician educator (EM) who had extensive experience in designing, implementing, and evaluating weight management curricula. The study team was assisted by four clinical provider expert consultants with experience in WMC. First, the study team created five vignettes intended to elicit each of the 5As (Ask, Advise, Assess, Assist, and Arrange). The 5As framework has also been used in prior trials to teach and evaluate the quality of patient-centered counseling [24] and WMC [25, 26]. Our teams prior work using the 5As framework showed they provided an efficient approach for busy clinicians seeking to counsel patients in brief encounters [27–29]. Next, we administered the vignettes to Internal Medicine residents from a University Medical School**,** including obtaining crowdsourced ratings from analog patients and creating and providing the personalized feedback which residents were able to view on the VCA. Lastly, we conducted debriefing interviews with the residents who completed the VCA to obtain their feedback on the vignettes and personalized feedback. This study was approved by the Institutional Review Board at the University Medical School.

#### Phase 1: vignette development

**Fig. 2:**
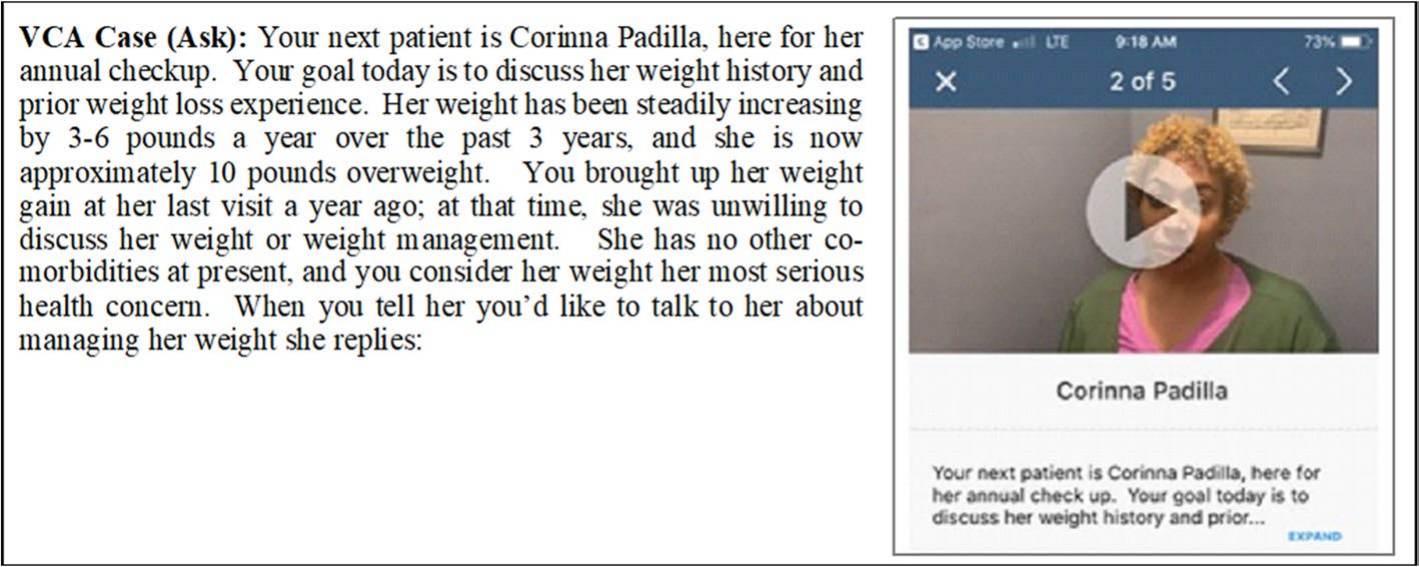
Sample VCA case

First, the study team drew on their prior trial examining the effect of a multi-modal WMC on counseling skills for health behavior change in medical students [30]. The prior trial compared students receiving the multi-modal WMC curriculum to those receiving traditional education on outcomes of a 23-item checklist integrated into the 5A framework based on the 2013 Guideline for the Management of Overweight and Obesity in Adults published by The Obesity Society and American College of Cardiology/American Heart Association [31]. We first highlighted checklist items that fewer than 50% of the intervention students performed during a study assessment. For example, only 24.6% discussed weight history and prior weight loss experience with their patient. Once this list was created, the team then solicited feedback from four external clinical providers with extensive experience in WMC. With their feedback, the team came to consensus on the 5 essential behaviors (of the original 23) to assess in patient scenarios, including one for each of the 5A constructs (see Results). The team then received training in how to create the description of the clinical situation, and patient script. Two research team members each created one of the five vignettes, which were reviewed and revised by the entire team. We solicited feedback from the Program Director for the Primary Care Track in the Internal Medicine residency program (EM) at the University Medical School in the process. Once vignettes and scripts were finalized, we recruited a diverse group of actors to portray the vignettes and videotaped them. The actors were portrayed in the vignettes multiple times, changing their voice tones, facial expressions etc. The team reviewed the videos and selected the ones that would be the most appropriate for the residents. Figure 2 shows the sample VCA case.

#### Phase 2: VCA pilot testing

##### Administration of the VCA

The Internal Medicine residency program director emailed all third year Internal Medicine Residency students and the post-doctoral fellows (*N* = 56) enrolled in the Fellowship programs at UMass Medical School. The email introduced them to the study through a Fact Sheet and asked for voluntary participation. Two reminder emails were sent to non-respondents. Residents and post-doctoral fellows who volunteered were provided detailed instructions on how to log on to a dedicated website or download the freely available National Board of Medical Examiners (NBME) VCA app on a smartphone or tablet. Participants who completed the VCA pilot were provided a $50 gift card to compensate them for their time.

##### Crowdsourced ratings and comments

We used the Amazon Mechanical Turk platform [32, 33] to recruit analog patients to review, rate, and comment on the responses. Analog patients are untrained lay people who are asked to rate their impressions of a medical interaction while imagining themselves as the patient in the encounter [34]. They are often used to assess patient perceptions when using traditional patients is unethical or impractical due to high costs and lack of time [32–34]. Analog patients have been shown to produce reliable ratings of physician communication skills using the VCA [35].

Each resident response was rated by 10–22 analog patients for five items, with the stem of “I would feel this provider…” 1. Understood how I was feeling, 2. Cared about me, 3. Respected what I had to say, 4. Listened carefully to me, and 5. Explained things in a way I could understand. Response options included: 1) Not at all 2) A little 3) Somewhat 4) Very much 5) Completely. A final rating question asked: “Overall, this provider’s response was:” with response options being 1) Poor 2) Fair 3) Good 4) Very Good 5) Excellent. After rating the last response in the set, the analog patient was prompted to answer a single open-ended question: “What would you want the provider to say if you were the patient in this situation?” We calculated individual means and standard deviations from these 6 questions of the resident’s response for each vignette generated from the analog patients’ rating, along with the cohort’s mean and standard deviations. We also calculated their overall score (mean score across all 5 vignettes), and the cohort overall mean. Quantitative ratings were created by averaging the 6 Likert scale questions; thus, the continuous scores were derived from ordinal approximations of continuous variables.

##### Creation of feedback reports

**Fig. 3:**
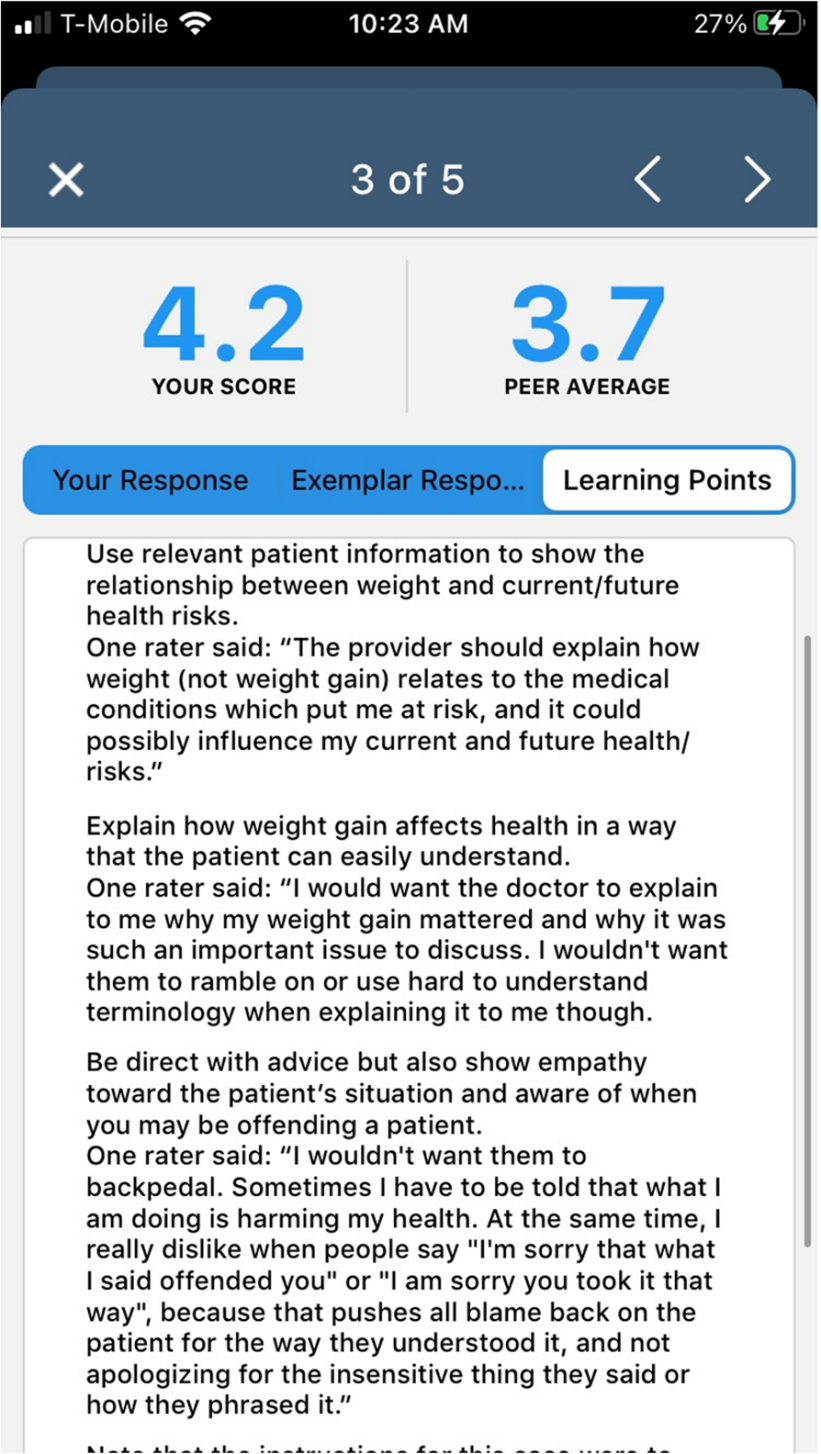
Sample feedback report

Based on analog patient ratings, the team reviewed the top three resident responses for each vignette and selected an exemplary response to be included in the feedback reports. We then generated actionable recommendations called “Learning Points”, on how to communicate effectively in the specific situation portrayed in the vignette, drawing heavily on the analog patients’ feedback on what they would have liked the resident to have said [36]. The final VCA feedback reports included three key elements: 1) analog patient ratings summarized as described above, 2) access to the audio recording of the exemplary response, and 3) “learning points” for each vignette based on careful review of the analog patients’ comments on what they would have wanted the provider to say, curated by communication experts to ensure appropriateness and clarity. The residents own response was also included in the feedback report to allow for comparison, and the original vignette text and video was available as context if desired. Residents received this report within 4 weeks of completing the VCA. Figure 3 depicts a sample VCA feedback report.

#### Phase 3: resident debriefing interviews

We conducted semi-structured telephone interviews with participants who completed the VCA training with the goal of exploring residents’ use and perception of the VCA and their implementation into practice. The interview guide was developed iteratively with qualitative experts and VCA developers on the team. Residents who completed the training were sent an email invitation. Interviews were approximately 30-min in length, conducted by a research team member (see Additional file 1). All participants provided verbal consent prior to completing the interview. Participants who completed interviews were provided a $50 gift card to compensate them for their time.

##### Analysis

All quantitative data presented were calculated using R version 4.1.0, including means and standard deviations of the analog patients’ ratings for each individual user and the entire cohort by vignette. For creation of the learning points and selecting exemplar responses, research team members were oriented to the process previously described by Mazor et al. [36]. After analog patients’ written responses to the open-ended question were downloaded into an Excel spreadsheet, unusable responses (blank or irrelevant statements) were removed from the analysis. Two research team members were assigned to each vignette to develop learning points based on the previously used approach of thematic analysis [37]. This involved generating codes and applying them to the data, using an iterative, inductive process to generate more codes as the data was analyzed. We generated themes from the data and selected illustrative quotes to represent each theme. Learning points and illustrative quotes were collated and presented to the entire research team for refinement, and to ensure that these were consistent with known best practices. Exemplar responses were selected from user responses that had the highest mean analog patient ratings by vignette.

All debriefing interviews were transcribed verbatim. We used thematic analysis to generate and apply codes, followed by identifying themes and illustrative quotes representing each theme. Four research team members each reviewed subsets of the transcripts in full, then, using open-ended coding, assigned codes/themes to segments of the text. Team members then met to conduct coding checks to ensure inter-rater agreement and resolve any disputes in coding [38]. Once resolved, they discussed perspectives and themes that emerged from the interviews. Emergent themes related to residents’ perceptions of the VCA and implementation into practice arose. Those themes and illustrative quotes are outlined in detail.

## Results

Of the 56 eligible residents, twenty-one initiated the VCA. Of the twenty-one, sixteen completed the full VCA pilot. Of the 16, eleven were male (68.8%) and 5 were female (31.2%). All participants were Internal Medicine categorial residents.

### Ratings, learning points and exemplar responses

The characteristics of the analog patient raters are presented in Table 1. More than half were males (*n* = 129, 57%) and higher educated, holding a Bachelor’s degree or higher (*n* = 183, 81%). The overall mean rating of residents’ responses to the vignettes from the analog patients was 3.7 (standard deviation 0.2, range 3.3, 4.1). By 5A construct, the means ranged from 3.55 to 3.85 on a scale of 1 to 5 (see Table 2). The learning points and illustrative quotes by vignette (and 5A construct) are also shown in Table 2. Analog patients provided recommendations on how they would like the provider to respond. Some also provided direct language on what the provider should say to someone in that scenario.

**Table 1:** Analog patient rater characteristics (*N* = 226)

Variable	n	%
Age		
18–24	6	2.7
25–34	95	42
35–44	68	30.1
45–54	35	15.5
55–64	14	6.2
65–74	8	3.5
Language		
English	224	99.1
English and another language	2	0.9
Gender		
Female	97	42.9
Male	129	57.1
Education		
Associate degree	13	5.8
Bachelor’s degree	143	63.3
Graduate degree	40	17.7
Graduated high school or equivalent	9	4
Some college, no degree	21	9.3

**Table 2:** Overall mean (SD) ratings and sample learning points and illustrative quotes by vignette/5A construct

Vignette/5A Construct	Mean (SD) ratings^a^	Sample learning points and illustrative quotes
**Ask** about her weight history and prior weight loss experience	3.55 (0.94)	Ask about the patient’s past experiences with weight loss, including what they tried and what worked *“I would want them to lay out some possible choices and procedures we might be able to take to reduce my weight after discussing what I've already tried.”*
**Advise** that weight loss is recommended based on the patient’s personal health information (e.g. BMI & risk factors)	3.70 (0.98)	Use relevant patient information to show the relationship between weight and current/future health risks *“The provider should explain how weight (not weight gain) relates to the medical conditions which put me at risk, and it could possibly influence my current and future health/risks.”*
**Assess** the patient’s level of motivation and confidence to make lifestyle changes	3.85 (0.91)	Gain understanding of the patient's past attempts at weight loss *“I would love it if the provider asked me the steps I have been taking, what I find hard or easy.”*
**Assist** the patient in setting goals and specific plans within the framework of providing relevant information regarding the relationship between weight, diet, and physical activity	3.72 (1.02)	Listen carefully to patients’ challenges/concerns and provide options to address the challenges that will help them meet their goals *“I know I'm overweight, so other than stating the obvious things, I would like them to talk to me about things I might not be aware of, like the one who talked about biking and that taking strain off the knees, or the water aerobics classes. Throwing out some of these examples gives me more hope that I can work towards my goals without just saying diet and exercise.”*
**Arrange** follow-up as appropriate to support her in her efforts to manage her weight	3.78 (0.93)	Show caring, warmth, empathy *“I'd want to hear [the doctor being] empathetic, warm, and taking time to point out it was a journey that they, too, cared about me taking”*

^a^Ratings were created by averaging the 6 Likert scale questions

### Qualitative feedback from VCA debriefing

Of the 16 participants completing the VCA, 11 completed debriefing interviews. We identified five themes regarding resident experiences with the VCA and application to practice. We present the themes and illustrative quotes below:

#### Theme 1: Overall the VCA was easy to use, helpful and more engaging than traditional learning and assessment modes

Participants noted the VCA was more engaging and provided additional ways to learn outside of the usual methods.

“VCA is a very cool app. And it's a nice, interesting way of being more engaging than us reading through PowerPoint slides.”

“And in medical school we have written exams, but there's not many oral exams really, or low pressure things like this was, because I could record again, so it was very low pressure.”

Participants noted the ease of using the app and liked being able to do it at their convenience.

“I thought the app was really easy to use. I honestly woke up, I made breakfast and then I just did it while I was eating breakfast and it was super easy.”

#### Theme 2: Patient scenarios were similar to those encountered in the clinic, including diversity, health literacy and different stages of change

Participants noted the similarities in the VCA to what they actually see in the clinic.

“I thought it was realistic. Like everything that happened in those case scenarios is something that I've encountered in clinic.”

Participants discussed the physical and psychosocial diversity presented in scenarios, including patient’s readiness to change and motivation levels.

“I think you had a good range of people. I mean, different demographics and also different stages of change and different kind of understanding of what's going on with their health and health literacy as well.”

“…and you had some diverse reactions to things, which I thought was pretty interesting just because it's not all just people who are motivated or positive. There are people who are defensive... So it was a nice spectrum of how people could respond..”

#### Theme 3: The knowledge, skills, and reminders from the VCA can be transferred to practice

Some residents intended to implement their new knowledge and skills into their own WMC practices with patients.

“I think the fact that you are providing an ideal response, you actually have something that you're using, you're getting the tone of voice, you're getting everything in there and then you can just copy that if you want to and implement that in your own practice.”

“I'll definitely bring it up to my patients more knowing how it should be done because I know it's a very sensitive topic for some people and just having this guideline really helps bring up the topic to patients. Just knowing how people appreciate it being done.”

“I had the opportunity to think about what I wanted to say without the pressure of somebody sitting there in front of me. I think it was helpful in the sense that weight can be a very uncomfortable topic for anybody. So just having the opportunity to think about it before I say something gives me like a little bit of a rubric that I can spring off of next time that it's in-person.”

#### Theme 4: Feedback reports were helpful, to the point and informative, including the exemplar response of how to best respond to the scenario.

Participants noted that getting feedback on the ideal response and what the patient wanted to hear was very helpful.

“I enjoyed how every patient there was a bullet of exactly what an ideal response would be. I felt like I made it more purposeful because I think that feedback is one of the best ways that we learn in that… having deep feedback as detailed as it was, was important. You can see that bar graph at the top that compared you to your peers so you get a better understanding of how well or how poorly you did not that that was the point of this exercise, but if you just get an idea of how much, or how little you need to refine your skills. I actually really enjoyed the way the format was. They hit the strong points without rambling too much”

“I thought it was helpful at the very end where it was kind of like the highlights of what the patient would have wanted to hear. I think those little nuggets are the most helpful.”

#### Theme 5: The VCA provides alternative and practice scenarios to real-life patient situations when they are not always accessible

Participants made mention of appreciating the clinical encounters when they were not actually able to be in the clinic or practice with real patients.

“Especially if we're in a situation where now there's like more EA learning and you know, the students are not going to have those patient encounters through OSCEs or however they were used to doing them. I think this is a great way to work around that.”

“With COVID we miss a lot of our clinic time, so I felt like I missed a big chunk of it. And just to have something to refresh me about how things are done in the clinic is really good.”

“...you do get to kind of do a dry run of what you want to say in a whole bunch of different scenarios before you actually speak to a patient…”

## Discussion

This study demonstrates the VCA is a promising tool for supporting residents in learning weight management counseling. Residents responded favorably, indicating the VCA is usable and acceptable. Different learning styles may benefit from the use of multi modal techniques, as noted by the residents’ mention of the VCA being more engaging than traditional methods of PowerPoint slides. Residents also felt the assessments were “low stakes”, providing unique opportunities to improve their skills through practice without being penalized.

Analog patients are becoming more widely used in research and are a valuable source of bringing the patient’s voice into the clinical encounter [39]. Responses of the analog patient raters underscore patients’ desire for empathy, understanding, compassion and caring in addressing their weight concerns. This is consistent with Mazor and colleagues’ findings of patients offering advice for how providers can empathize with patients [36]. For example, analog patients stated, “I'd want to hear the doctor being empathetic, warm, and taking time to point out it was a journey that they, too, cared about me taking.” Prior health interventions have found that expert-written messages were more “biomedical” (avoidance, behavioral strategies, health), while peer messages had more “social” and “real-life” content (expectations, money, quality of life, attitudes, and friends) [40]. Providing lay language from analog patients on how to communicate may help providers resonate with patients as they can reflect shared experiences and possibly address sociocultural characteristics [41]. One analog patient touched upon shared experiences with providers, “…if the provider used more language to reaffirm how I was feeling or perhaps even references their own struggle at times…”.

During the vignette development process, we aimed to provide a diverse sample of patients and clinical scenarios. Residents’ comments in the interviews suggest we achieved this (i.e., “…you had a good range of people…different demographics, different stages of change and health literacy …you had some diverse reactions to things”). Preparing medical residents to encounter diverse patient populations is critical. Racial and ethnic disparities have been noted in obesity and chronic health conditions – Non-Hispanic Black and Hispanic adults have the greatest prevalence of obesity in the US [42]. Racial and ethnic differences are associated with higher mortality rates from diabetes, cardiovascular disease, and cancer [43]. There is a need for culturally sensitive approaches to weight management interventions [44]. The opportunity to practice communicating with patients from different backgrounds via the VCA may provide opportunities to tailor exemplar responses to contextual factors for patient scenarios and characteristics.

COVID-19 presented a challenge to residency training programs across the US. Residency directors moved classes to online formats, restricting site rotations, and limiting in-person gatherings in order to reduce patient contact and reduce the spread of the virus [45, 46]. Though the VCA technology was developed prior to COVID-19, it can help to address these challenges. While WMC is the focus of our trial, prior work using the VCA has included other patient scenarios, including medication adherence, antibiotic use, medication costs and recent medical diagnoses [36]. Given the customizable nature of the VCA tool, it presents opportunities for other clinical scenarios to be presented and skills practiced. A common theme that emerged from resident interviews was the knowledge, skills, obtained via the VCA is transferable; many residents volunteered that they intended to implement what they had learned into their own WMC practices. Several behavioral theories suggest that behavioral intentions predict subsequent behavior [47–49], including in healthcare professionals [50]. Given that many moderating factors and complexities of patient encounters may lessen the ability to predict actual behavior, vignettes have been used to address this. Prior work using vignettes to predict prescribing behavior amongst physicians explained 26.8% of variance in behavior assessed objectively [51], and vignettes may hold promise in increasing physicians behavioral performance and subsequent behavioral intentions [50].

This study has limitations. As noted in Mazor et al. [22], future trials need to assess whether VCA scores are correlated with other measures of clinician-patient communication, patient experience, or patient satisfaction, potentially through patient exit interviews. It is also necessary to assess whether practice and feedback using the VCA results in improved provider performance in delivering effective WMC to patients in routine clinical care. We see these as next steps in a large-scale randomized trial implementing VCA training into medical education curriculum. Further consideration needs to be given to examining the associations between analog patient characteristics, including gender, race, ethnicity, education, and weight status, and their ratings of responses. Like prior work using the Amazon Mechanical Turk platform to recruit analog patients [36, 39, 52–55], our analog patients had high education levels. We were also unaware of the weight status of our analog patients, thus they may not represent a population with overweight and obesity. However, recent data show analog patient responses to two patient communication vignettes (cancer and obesity) from analog patients with those conditions were highly correlated to responses from analog patients without those conditions [56]. While the VCA has the option to restrict analog patient raters to meet prescreening patient criteria, analog patient responses and ratings from those without pre-specified health conditions may serve as proxies, but further research is warranted. We also were unable to conduct comparisons of provider characteristics on preferences for use of the VCA for WMC, despite prior evidence that female physicians have displayed less negative attitudes toward WMC than their male counterparts [57]. With our qualitative interview sample consisting of 69% males showing a high overall level of acceptability for WMC training via the VCA, future larger trials may investigate gender differences. Lastly, we created patient vignettes addressing provider 5A behaviors we observed were lacking (< 50%) in our prior trial, but other behaviors may need to be addressed or frameworks studied.

## Conclusion

We demonstrated the feasibility and acceptability of the VCA, a technology delivered platform, to deliver WMC to medical residents. Our use of analog patients to review and respond to residents WMC responses allowed us to generate learning points for each of the 5A constructs our vignettes addressed. Further, residents noted appreciating the diversity and range of patient experiences to which the VCA exposed them, providing potential opportunities to tailor providers’ responses to sociological and cultural factors in WMC scenarios. As medical evidence is constantly evolving, several organizations, including the Institute of Medicine and the Obesity Medicine Education Collaborative, recommend WMC training beyond medical school to reinforce and extend prior training [58, 59]. The VCA would be a novel and convenient approach to provide this training. Future work will examine the effect of the VCA on WMC in actual clinical practice.

## Supplementary Information

**Additional file 1. **Interview questions for VCA debriefing.

## Data Availability

The datasets used and/or analysed during the current study are available from the corresponding author on reasonable request.

## Abbreviations

NBME: National Board of Medical Examiners
WMC: Weight management counseling
VCA: Video Communication Assessment
