# Supplementary material for: Video-based communication assessment for weight management counseling training in medical residents: a mixed methods study
Source: BMC Med Educ. 2022 Dec 28;22:899. doi: 10.1186/s12909-022-03984-6 (PMC9795434; doi:10.1186/s12909-022-03984-6)
Supplement: Supplementary file 1 — Additional file 1. Interview questions for VCA debriefing. [file 12909_2022_3984_MOESM1_ESM.docx]

**Additional file 1. Interview questions for VCA debriefing.**

| 1. What did you think of the VCA overall?    1. Was there anything else you thought of that you didn’t already mention? 2. How similar or different were the patient scenarios to the patients you care for?    1. Are there are other weight management scenarios you have encountered that might be useful to include? 3. What one new thing did you learn as a result of completing the VCA program?    1. How did the VCA guide you to think about your weight management counseling skills (or these scenarios)? 4. Would you recommend this tool to your colleagues? Why or why not?    1. What could be improved to make this a more useful tool? 5. What did you think about the feedback report?    1. What did you find most useful about it?    2. How might the report be improved? 6. What kind of device did you do the VCA on? Did you have any trouble downloading or navigating through it? 7. Is there any additional feedback regarding the feedback report of VCA overall you would like to share? |
| --- |
